# Endoscopic negative pressure therapy (ENPT) of a spontaneous oesophageal rupture (Boerhaave’s syndrome) with peritonitis – a new treatment option

**DOI:** 10.1515/iss-2020-0043

**Published:** 2021-04-06

**Authors:** Gunnar Loske, Katrin Albers, Christian T. Mueller

**Affiliations:** Department for General, Abdominal, Thoracic and Vascular Surgery, Katholisches Marienkrankenhaus Hamburg gGmbH, Hamburg, Germany; Clinic for Anaesthesiology, Pain Therapy and Intensive Care, Katholisches Marienkrankenhaus Hamburg gGmbH, Hamburg, Germany

**Keywords:** Boerhaave, drainage, endoscopic vacuum therapy, endoscopy, oesophagus, perforation, peritonitis, nasogastric tube

## Abstract

**Objectives:**

Boerhaave’s syndrome is a life-threatening disease with high mortality and morbidity. Endoscopic negative pressure therapy (ENPT) can be used to treat oesophageal perforations.

**Case presentation:**

We report on a case of oesophageal rupture with peritonitis in a 35-year-old male patient. The start of treatment was 11 h after the perforation event. The treatment of the perforation defect was performed exclusively by intraluminal ENPT, the treatment of peritonitis was performed by laparotomy with abdominal lavage. For ENPT we used two different types of open-pore drains. The first treatment cycle of four days was performed with an open-pored polyurethane foam drainage (OPD), which was placed intraluminal to cover the perforation defect and to empty the stomach permanently. The second treatment cycle of nine days was performed with a thin nasogastric tube like double-lumen open-pored film drainage (OFD). For suction OPD and OFD were connected with an electronic vacuum pump (−125 mmHg). OFD enables active gastric emptying with simultaneous intestinal feeding via an integrated feeding tube. Intraluminal ENPT with a total treatment duration of 13 days was able to achieve the complete healing of the defect. Surgical treatment of the perforation defect was not necessary. The patient was discharged 20 days after initial treatment with a non-irritating abdominal wound and a closed perforation.

**Conclusions:**

In suitable cases, endoscopic negative pressure therapy is a minimally invasive, organ-preserving procedure for the treatment of spontaneous oesophageal rupture.

## Introduction

Spontaneous oesophageal rupture caused by vomiting and frequently associated with alcohol intoxication (Boerhaave’s syndrome) is a life-threatening disease with high mortality and morbidity [1]. Usually, the defect is located in the distal oesophagus at the gastroesophageal junction and thus contamination by the escaping secretion results in mediastinitis. Less frequently, the defect opens into the abdominal cavity, causing subsequent peritonitis [2]. Endoscopic negative pressure therapy (ENPT) is a new suitable method for the treatment of all types of oesophageal defects [3, 4]. ENPT can be used to treat Boerhaave’s syndrome [5]. We report on a case of a spontaneous rupture with peritonitis.

## Case description

### Diagnostic tests

A 35-year-old male patient presented with persistent severe epigastric pain lasting 8 h. The complaints had developed after severe alcohol-induced vomiting with symptoms of reflux. During the physical examination, it was possible to elicit peritonitic tenderness throughout the entire abdomen. The laboratory inflammation parameters were significantly elevated. Since the perforation of a hollow organ was clinically suspected, we immediately performed an abdominal CT. This showed inflammatory wall-thickening with free intra-abdominal air and air inclusions at the gastroesophageal junction. Consistent with the present findings and under the suspicion of a Boerhaave’s syndrome, we intended to perform oesophagogastroscopy with CO_2_ insufflation under anaesthesia in the operation room. The endoscopy was expected to precisely locate the perforation site with a description of the local conditions (size, perfusion). An indication for endoscopic treatment was also to be evaluated. After this, laparoscopy was planned for evaluation of the abdominal cavity.

**Figure 1:**
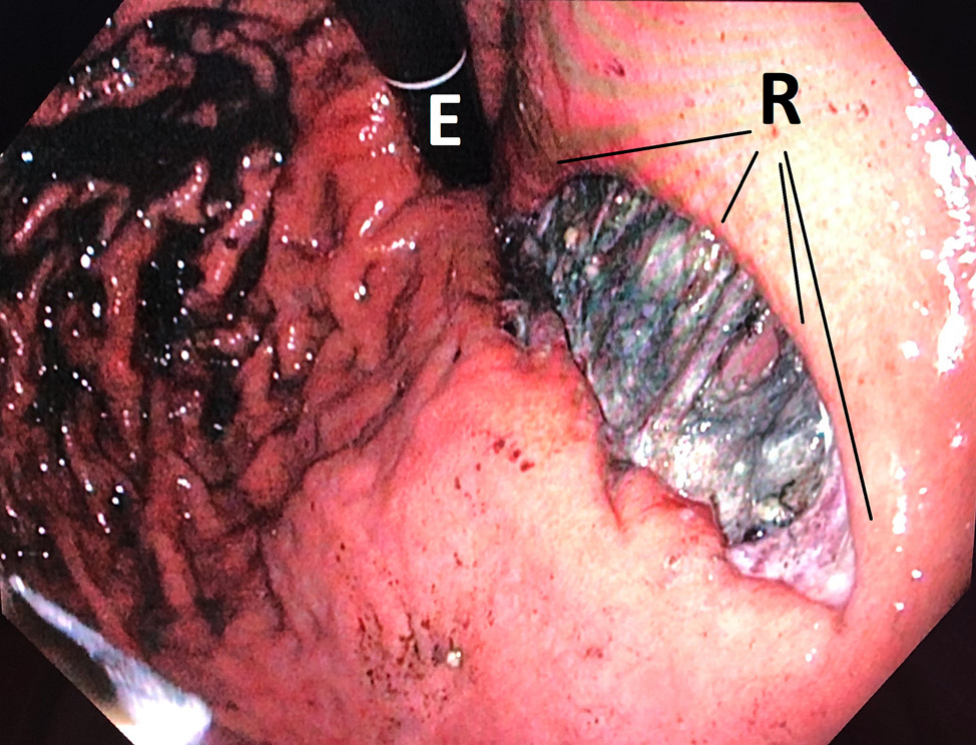
Endoscopic intraoperative presentation of the perforation defect of the rupture (R) in the gastro-oesophageal junction in a hiatal hernia. The endoscope (E) is in inverted rotation.

Flexible endoscopy detected a blackish, cloudy secretion in the stomach which extended into the distal oesophagus. Erosive oedematous swelling could be seen in the distal oesophagus with an associated hiatal hernia and hiatal insufficiency. The deep and approximately 5 cm long by 2 cm wide, profoundly gaping rupture was found at the distal gastroesophageal junction (Figure 1). The blackish coated muscle fibres were exposed extensively. The visible transmural defect in the abdominal cavity could not be imaged endoscopically. Perfusion in the margins of the defect was normal. We intentionally did not perform endoscopic manipulation at the perforation, such as probing with an instrument.

In accordance with our algorithm for treating oesophageal defects [5], we decided that initiating intraluminal endoscopic negative pressure therapy (ENPT) was indicated. This should cover the defect and initiate long-term emptying of the stomach.

Treatment of the Boerhaave defect with endoscopic negative pressure therapy (ENPT).

## Materials and methods

**Figure 2:**
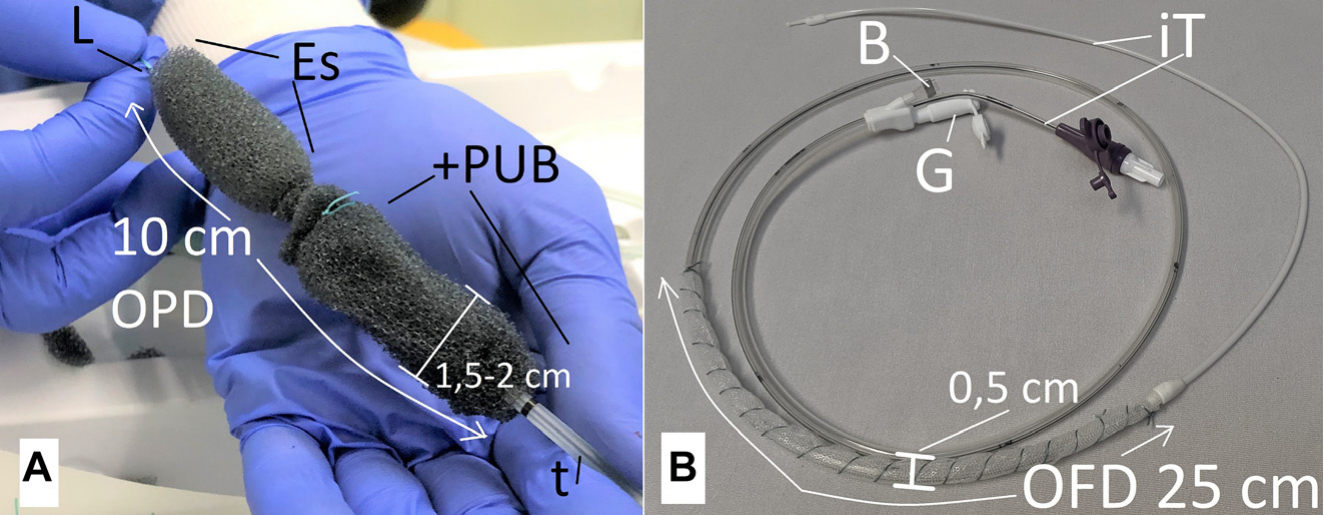
Two types of open-pore drains were used for intraluminal negative pressure therapy. A: 10 cm long open-pore polyurethane drainage (OPD), diameter of 1,5-2 cm, an open-pore polyurethane foam body (+PUB) was attached to an Esosponge^®^ drainage (Es), loop (L) was fixed at the tip of the OPD, drainage tube (t); B: open-pore film drainage (OFD) with a 25 cm long open-pore drainage element consisting out of open-pore film which is wrapped surround the gastric channel of a Freka^®^ Trelumina, diameter 0.5 cm; ventilation channel is blocked with a clamp (B), intestinal feeding tube (iT), adapter (G) of the gastric tube to which the vacuum pump is connected.

For intraluminal ENPT we used two different types of drains: an open-pore polyurethane foam drain (OPD) (Figure 2A) and a double-lumen open-pore film drain (OFD) (Figure 2B).

In the first ENPT cycle, we used OPD with an approximately 10 cm-long, polyurethane foam body (PFB)of 1.5–2 cm in diameter (Figure 2A) [3, 4, 6]. This size was considered to be adequate to cover the entire defect area with certainty and to extend beyond its margins both proximally and distally. Since such drainage is unavailable as a medical device, we modified an Eso-SPONGE drain (Eso-SPONGE^®^, B. Braun). First, the drainage tube was given two additional lateral perforation openings proximal to the PFB. We then attached an additional approx. 6 cm long PFB around this drainage tube section. The polyurethane foam used was the foam material from an Endo-SPONGE drain (Endo-SPONGE^®^, B. Braun, Melsungen, Germany). The distal end of the OPD was finally given a suture loop which could be grasped with endoscopic forceps.

**Figure 3:**
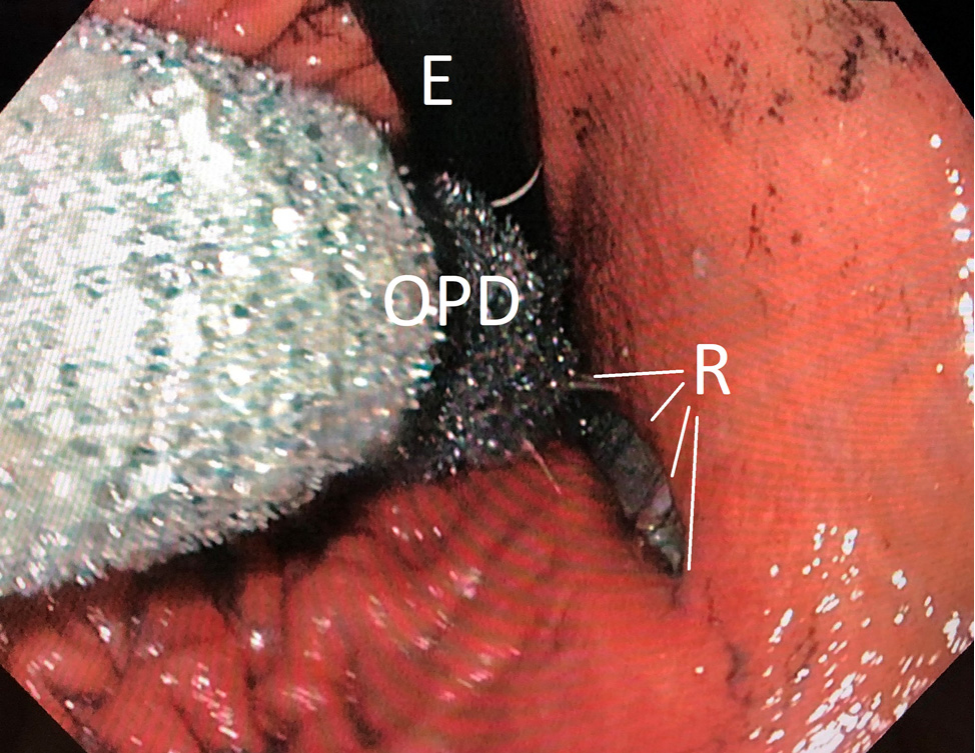
Endoscopic insertion of the long open-pore polyurethane foam drainage (OPD) in the gastrooesophageal junction in order to cover the defect (R) completely. Endoscope (E).

After grasping the suture loop, the OPD with the 10 cm-long PFB was introduced orally into the oesophagus under permanent endoscopic monitoring and advanced into the stomach. The loop was opened, and the 10 cm-long foam body was placed intraluminally in gastro oesophageal junction and the oral stomach, covering the large defect zone completely (Figure 3). Continuous negative pressure of 125 mmHg was applied to the OPD using an electronic pump (KCI V.A.C. Activac, KCI USA Inc., San Antonio, Texas, USA, setting – 125 mmHg, continuous, intensity 10).

**Figure 4:**
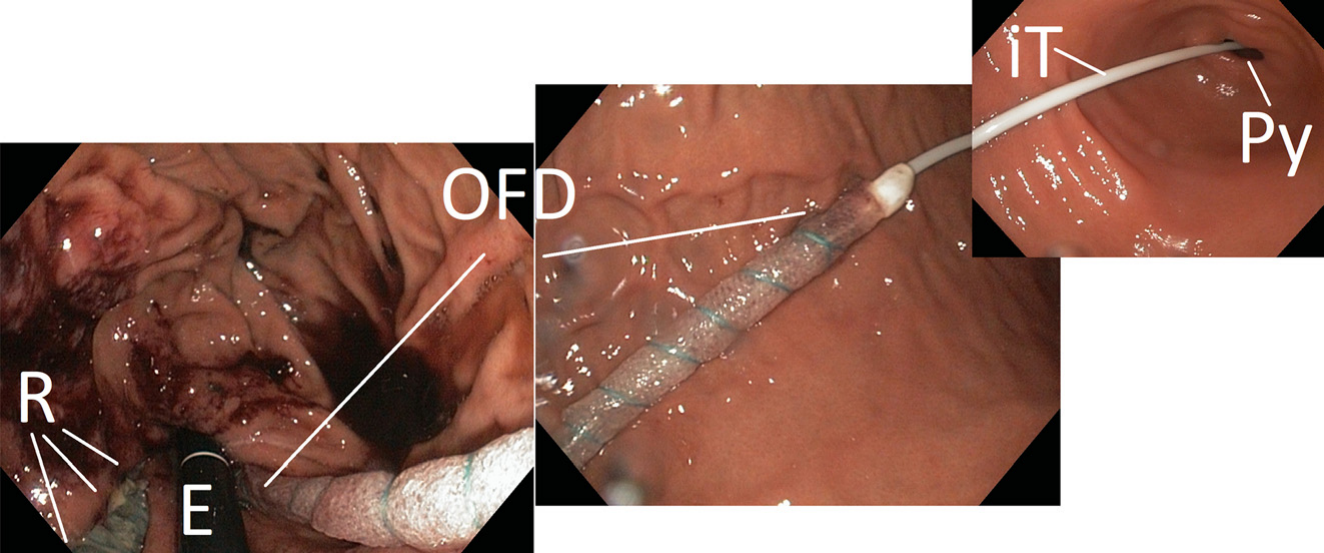
Demonstration of the endoscopic placement of the NGT-like OFD with a 25 cm long drainage-film element over the entire length of the stomach. The intestinal feeding tube (iT) lies in the pylorus (Py). The endoscope (E) and the rupture (R) can be seen in inversion position.

For the subsequent intraluminal ENPT treatment cycles, we used a new double-lumen, open-pore film drainage (OFD) (Figure 2B) [6]. A 3 cm narrow strip of an open-pore, double-layer drainage film (Suprasorb^®^ CNP-Drainage Film, Lohmann & Rauscher, Rengsdorf, Germany)was used to wrap the lateral perforation openings of a Trelumina tube (Freka Trelumina, CH/Fr 16/9, 150 cm; Fresenius, Germany) in a single layer. The thus designed drainage element was 25 cm-long. The film was originally developed for use in abdominal negative pressure therapy and consists of a double-layer, thin, perforated membrane. Fluids can be conducted within and through the membrane. The gastric lumen of the double-lumen OFD was dedicated to the active negative pressure drainage of the stomach. It was connected to the electronic negative pressure pump and a negative pressure of 125 mmHg was applied as before. The second channel is equivalent to the intestinal feeding tube; it was inserted into the duodenum. Simultaneously to the intraluminal ENPT of the stomach, it was used for enteral nutrition. This thin OPD has a diameter of only 5 mm. It is introduced transnasally like a nasogastric tube (NGT) and correctly positioned under endoscopic monitoring (Figure 4). OFD and OPD were fixated with a suture to the nasolabial fold. This is intended to prevent dislocation through propulsive peristalsis or respectively iatrogenic removal.

The open-pore drains were in each case changed after three to four days and the course of internal wound healing was evaluated. Removal for the change was done by pulling the drainage tube after disconnection from the pump.

### Surgical therapy of the peritonitis

Due to the clinical presentation of an acute abdomen with radiological detection of free air, laparoscopy was indicated and performed immediately after endoscopic inspection and initiation of ENPT. We found peritonitis affecting all four quadrants with already adhering deposits. The procedure was expanded to a median epigastric laparotomy in order to perform adequate lavage of all spaces with removal of the secretions and deposits. The perforation site was not surgically treated. The procedure was ended with the placement of passive drains and a primary closure of the abdominal wall.

## Results

Endoscopy and laparoscopy were performed 11 h after the first symptoms. The combined procedure of endoscopy with ENPT and laparotomy lasted a total of 80 min.

Intraluminal ENPT with a total treatment duration of 13 days was able to achieve the complete healing of the defect. After placement of negative pressure on the OPD and OFD, it was possible to endoscopically observe the complete collapse of the gastric lumen. The liquid digestive secretion of the stomach was permanently extracted in an active manner with suction. Under negative pressure drainage, it was possible to observe the collapse of the wound surface and adaptation of the wound margins. During the endoscopic follow-up examinations and changing of the drainage, the stomach was completely empty by the negative pressure in all cases.

The first cycle of four days duration was performed with the 10 cm-long OPD which was placed in the oral stomach and gastroesophageal junction in a manner to cover completely the perforation defect. During the first replacement of the drainage, the contact sites on the mucosa exhibited typical erosive suction effects. However, the foam body had not been extracted to the wound surface. At the first exchange the wound was still gaping and exhibited a blackish coating. It was noticeable at the fixation suture of the nasolabial fold that the OPD was under tension due to propulsion. The incising suture resulted in a pressure ulcer of the nasal wing which necessitated padding to relieve the suture.

Intraluminal ENPT was continued for nine days with the thin double-lumen NGT-like OFD with simultaneous enteral nutrition along the intestinal feeding tube. At intervals of three to four of days, the OFD was changed a total of three times. On the seventh day of treatment, granulating secondary wound healing could be seen at the base of the wound.

**Figure 5:**
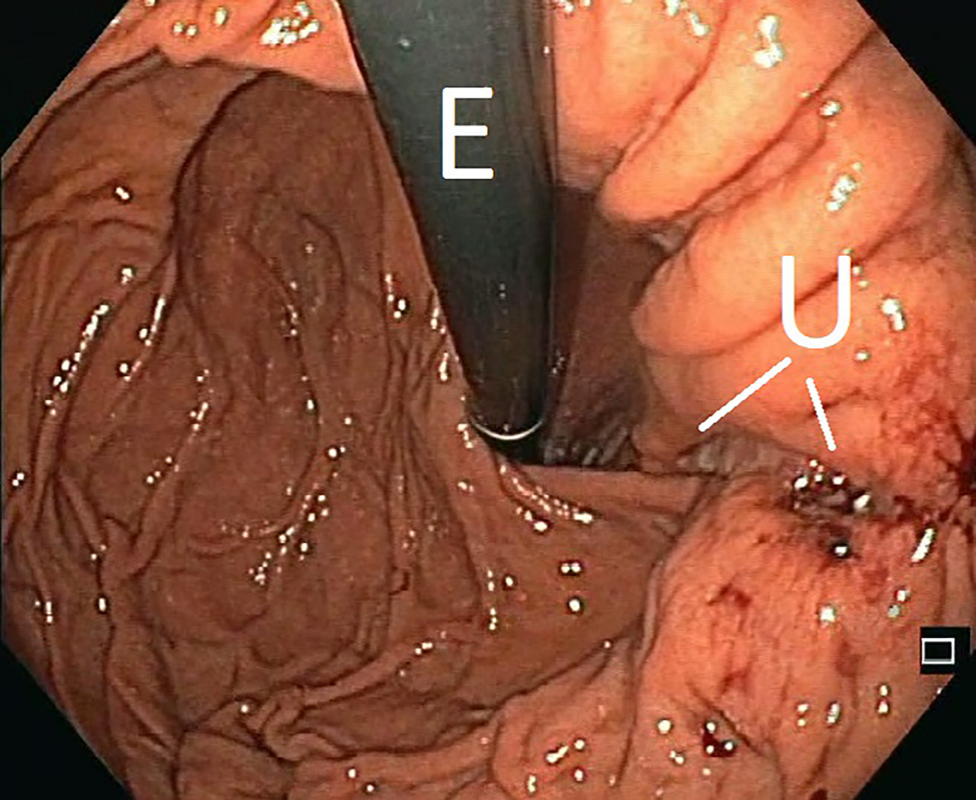
Final examination seven days after the end of the intraluminal endoscopic negative pressure therapy, the former defect is still recognizable as a narrow ulceration (U). Endoscope (E). With these findings the patient was discharged.

After a total of 13 days intraluminal ENPT, negative pressure therapy was terminated. The patient was extubated and the return to an oral diet, from liquid to normal food, was quickly undertaken. The endoscopic follow-up examination three and seven days after the end of ENPT therapy showed that of the former perforation only an small ulcer with a diameter of approximately 1 cm remained (Figure 5).

Due to postoperative withdrawal symptoms of delirium, it was necessary to sedate the patient while on intubation and ventilation for the entire duration of ENPT. The patient removed the OFD himself once and ENPT was interrupted for a few hours. A new OFD was inserted.

Calculated anti-infective therapy was initiated and adjusted consistent with the antibiogram of the surgically obtained smear. The septic course with initially high inflammatory parameters regressed under endoscopic therapy. Primary abdominal wound healing occurred and the surgical drainage could be removed after a few days as no secretion was produced any longer.

After being hospitalised for a total of 20 days, the patient was discharged with a non-irritating abdominal wound and a closed perforation.

## Discussion

The rupture of the distal oesophagus and the gastrointestinal junction triggered by vomiting is a surgical emergency with high morbidity and mortality. Boerhaave’s syndrome, first described in 1724, typically exhibits pulmonary symptoms [7]. However, the clinical presentation of the barotrauma is diverse and, as in our patient’s case, can also be associated with a perforation in the abdominal cavity and development of peritonitis. The immediate diagnosis and rapid initiation of treatment measures can be lifesaving. An essential prognostic factor is the time interval between the occurrence of the first symptoms and the first treatment measure [8].

A broad spectrum of surgical treatment options is available [9]. Distal defects can be sutured and covered by fundoplication. As a last resort, discontinuity resection with blind closure and a cervical salivary fistula is performed. Reconstruction takes place in two sessions, using gastric pull-up or an interposed colonic segment.

Various endoscopic techniques are available [10]. Clip systems and endoscopic suturing have the objective of closing the defect directly via a surgical suture. If the defect is at a proximal location, covered self-expanding stents are used for bridging and sealing. The simplest conservative method, which is considered in selected cases, consists of placing a NGT with the objective of removing the digestive secretion, thus interrupting contamination.

We present our new treatment concept, in which we use endoscopic negative pressure therapy [4, 5, 11, 12] and surgical treatment options in a complementary manner. Endoscopy is of great value, both diagnostically and therapeutically [13]. Endoscopy results in the exact imaging of the extent of the defect, the perfusion situation, and the location. Simultaneously, with the initiation of intraluminal ENPT, the therapeutic measure for treating the defect followed.

The CT initially performed yielded the decisive indication of an inflammatory process at the oesophagogastric junction with detection of free air and facilitated more precise planning of the treatment procedure before the operation [14].

In endoscopic negative pressure therapy, a distinction is made between an intraluminal variant, which is used as in our case, and an intracavitary extraluminal variant [15]. We have use drains made of an open-pored polyurethane foam (OPD) or an open-pored double-layer foil (OFD) which differ in their physical properties. Open-pore drains, both OPD and OFD [6, 16], are characterised in having multiple pores in open-pore contact, with each other. Even if several pores become obstructed or are suctioned to tissue, others remain open and suction fluids can be drained through these. The open-pore polyurethane foams have adjacently located pores. OPDs are voluminous and thus the endoscopic insertion manoeuvre can be somewhat more difficult, especially for long drainage elements. Overtubes are helpful. They secure access in the pharynx and the upper ooesophagus. One advantage of OPDs is the stronger adherence of the sponge material to tissue which facilitates a direct mechanical closure of the wound. On removal, typical erosive suction patterns can subsequently be observed, as was also the case for our patient. In our case, the sponge was not extracted onto the wound, but instead only led to the adaptation of the of the wound margins. In the OFDs, the pores are spaced further apart. Fluids can be transported through the double-layer film as well as within the two film layers. OFDs do not attach as strongly to tissue under extraction. In our case, we did not detect any extraction patterns on the mucosa. OFDs have the advantage of being very thin; placement is comparable to the easy manoeuvre required with a NGT. We used a new double-lumen OFD with an integrated feeding probe, which, under the negative pressure therapy in the stomach, allowed us to have simultaneous intestinal nutrition [17].

Using open-pore drains to which negative pressure is applied; it was possible to extract the liquid digestive and wound secretion out of the stomach permanently and completely. This interrupted the contamination of the wound surface and the extraluminal escape of secretion. The stomach collapsed and the wound margins were adapted. In our case, the endoscopic examination showed that after the tearing trauma, the defect was already covered by tissue again. A surgical suture or covering was therefore not necessary. In case of a visible defect opening into the free abdominal cavity, a surgical suture or covering would have been necessary in addition to the intraluminal ENPT. In our case study it was possible after initial laparotomy and endoscopy to continue the organ preserving treatment exclusively with the intraluminal endoscopic negative pressure therapy.

Conclusion: In suitable cases, endoscopic negative pressure therapy is a minimally invasive, organ-preserving procedure for the treatment of spontaneous oesophageal rupture. Its essential benefits include the effective and permanent elimination of the gastric digestive secretion. Newly developed double lumen vacuum drains enable simultaneous enteral nutrition with negative pressure therapy.
