# Supplementary material for: Endoscopic negative pressure therapy (ENPT) of a spontaneous oesophageal rupture (Boerhaave’s syndrome) with peritonitis – a new treatment option
Source: Innov Surg Sci. 2021 Apr 6;6(2):81–6. doi: 10.1515/iss-2020-0043 (PMC8435266; doi:10.1515/iss-2020-0043)
Supplement: Supplementary file 1 [file iss-06-20200043-s001.pdf]

Reviewer Assessment

G. Loske, K. Albers, C. Müller: Endoscopic Negative Pressure Therapy (ENPT) of a spontaneous oesophageal rupture (Boerhaave's syndrome) with peritonitis – a new treatment optionReviewers' Comments to Original Submission

Reviewer 1: Franzke, Tim

Date received: 11-Jan-2021
Reviewer recommendation: **Accept in present form**
Reviewer overall scoring: **High**

Assessment Form scores: 5 = High/Yes; 3 = Medium/Adequate; 1 = Low

Is the subject area appropriate for the journal	5				
Does the title clearly reflect the paper's content?	5				
Does the abstract clearly reflect the paper's content		4			
Do the keywords clearly reflect the paper's content?	5				
Does the introduction present the problem clearly?	5				
Are the results/ conclusions justified?		4			
How comprehensive and up-to-date is the subject matter presented?	5				
How adequate is the data presentation?				2	
Are units and terminology used correctly?		4			
Is the number of cases adequate?					1
Are the experimental methods/ clinical studies adequate?			3		
Is the length appropriate in relation to the content?		4			
Does the reader get new insights from the article?		4			
Please rate the practical significance.		4			
Please rate the accuracy of methods.					
Please rate the statistical evaluation and quality control.			3		
Please rate the appropriateness of the figures and tables.		4			
Please rate the appropriateness of the references.					
Please evaluate the writing style and use of language.			3		
Please judge the overall scientific quality of the manuscript.		4			
Are the methods used worthy of reproduction in greater deal?	Yes				

Would you be willing to review a revision of this manuscript?	Yes

Comments to author: Endosponge therapy in the esophagus has been an established method in my view for years. The effectiveness of this therapy has been confirmed by some series. The combination of a Trelumina nutrition probe with OPD is very interesting. However, there is a need for close interdisciplinary cooperation and individual strategy in each new case. The age of perforation is also crucial

Reviewer 2: Freys, Stephan

Date received: 16-Jan-2021

Reviewer recommendation: **Accept in present form**

Reviewer overall scoring: **Excellent**

Assessment Form scores: 5 = High/Yes; 3 = Medium/Adequate; 1 = Low

Is the subject area appropriate for the journal	5				
Does the title clearly reflect the paper's content?	5				
Does the abstract clearly reflect the paper's content	5				
Do the keywords clearly reflect the paper's content?	5				
Does the introduction present the problem clearly?	5				
Are the results/ conclusions justified?	5				
How comprehensive and up-to-date is the subject matter presented?	5				
How adequate is the data presentation?	5				
Are units and terminology used correctly?	5				
Is the number of cases adequate?	5				
Are the experimental methods/ clinical studies adequate?		4			
Is the length appropriate in relation to the content?		4			
Does the reader get new insights from the article?	5				
Please rate the practical significance.	5				
Please rate the accuracy of methods.	5				
Please rate the statistical evaluation and quality control.					
Please rate the appropriateness of the figures and tables.	5				
Please rate the appropriateness of the references.	5				
Please evaluate the writing style and use of language.	5				
Please judge the overall scientific quality of the manuscript.	5				
Are the methods used worthy of reproduction in greater deal?	Yes				
Would you be willing to review a revision of this manuscript?	Yes				

Comments to author: This is a well written case report of a novel technique to treat a rare but very important gastrointestinal surgical emergency.

The method is explained in detail, the results are well understandable, the clinical data are sufficiently described, and the discusssion is well balanced. The cited literature is up to date and fully appropriate, the figures demonstrating the new procedure are of excellent quality, well selected and understandable.

Being a single case report the overall scientific quality is very good.

This paper presents an innovative combined surgical-endoscopic interventional technique for a rare but extremely important surgical emergency. The authors reflect the expertise of a definitely very experienced group in this specific field. The fact that this is "only" a single case report may not compromise the high clinical practical value.
In summary: the classic case of an article reflecting a rather high clinical significance in lack of existing statistical significance.
In addition, the author can write a review about the innovation of his technique.
I suppose both case series and Review can be even published in journals with much higher impact
